# A unique inbred rat strain with sustained cephalic hypersensitivity as a model of chronic migraine-like pain

**DOI:** 10.1038/s41598-018-19901-1

**Published:** 2018-01-30

**Authors:** Gordon Munro, Steffen Petersen, Inger Jansen-Olesen, Jes Olesen

**Affiliations:** Danish Headache Center, Department of Neurology, Glostrup Research Institute, Nordre Ringvej 69, 2600 Glostrup, Denmark

## Abstract

Animal models of migraine-like pain enabling ongoing study of behaviour typically involve the systemic administration of chemical vasodilators or dural administration of inflammatory algogens. However, neither method mediates prolonged effects on behavior indicative of enduring pathophysiological changes occurring within dural or trigeminal pain circuits. We generated successive generations of a unique inbred rat strain, spontaneous trigeminal allodynia (STA) rats, previously reported to exhibit an episodic migraine-like behavioural phenotype. We show that both male and female STA rats display robust and sustained reductions in periorbital thresholds to cutaneous mechanical stimulation. Otherwise, the general behavior (e.g. locomotor, grooming) of these rats appeared normal. In female STA rats, the mechanical hypersensitivity was confined to the cephalic region, manifested after puberty through adolescence, and was sustained into adulthood recapitulating the clinical manifestation of migraine. We exploited this hitherto unidentified chronic phenotype to show that the migraine-specific drugs sumatriptan (5-HT_1B/1D_ receptor agonist) and olcegepant (CGRP receptor antagonist) could completely reverse cephalic hypersensitivity using a within subject cross-over paradigm. Our findings indicate that STA rats actually possess a phenotype indicative of migraine chronicity which is exquisitely sensitive to migraine therapeutics. This unique strain could prove to be an invaluable resource in preclinical migraine drug discovery.

## Introduction

Animal models of migraine-like pain enabling evaluation of disease-relevant behaviours, typically in mice or rats, are frequently induced by chemical provocation using systemic administration of vasodilating agents such as nitric oxide (NO) donors or dural application of inflammatory mediators^1–6^. These models have variously proven to be of some value in the preclinical setting but have their limitations^7^. Crucially, even with repeated dosing, the systemic administration of NO donors (e.g. glyceryl trinitrate; GTN or isosorbide dinitrate) in rodents has never been reported to produce sustainable cephalic or extracephalic hypersensitivity lasting longer than 1–2 weeks^2,8–10^, thereby limiting their utility in long term behavioural studies.

The use of genetic models of migraine-like pain in animals might be expected to partially mitigate such issues. Familial Hemiplegic Migraine is a rare monogenic migraine subtype associated with aura, characterized by weakness on one side of the body during the aura phase^11^. To date, the involvement of three genes has been confirmed in this migraine subtype of which the humanized knock in CACNA1A-related R192Q mouse is probably the best studied^12^. These mice display increased unilateral head grooming and are photosensitive in accordance with a migraine-like phenotype. An advantage of using such models is that the animals possess a pre-defined phenotype, circumventing the need for complicated dosing protocols using NO donors or surgical procedures (e.g. dural administration of inflammatory algogens), which can make replication of data between labs a trial in itself^7^. However, a downside is that the specific mutation involved might not be of relevance to common polygenic forms of migraine in the general population.

Recently, a rat model of spontaneous trigeminal allodynia (STA) with migraine-like features has been described^13^. Derived from a single male Sprague-Dawley (SD) rat which displayed a fluctuating periorbital pain threshold to cutaneous stimulation with von Frey filaments, the trait was transferred to subsequent generations of both sexes. By the tenth generation (F10), the behavioural phenotype (which included sensitivity to sound) was apparently expressed at 50–75%. Importantly, the cephalic hypersensitivity was sensitive to acute intervention with sumatriptan, prophylactic treatment with valproate, and could be provoked by GTN. Clearly, the general availability of such a model could revolutionize preclinical migraine research. However, a more extensive characterization by independent laboratories is imperative before it can be considered viable for such purposes. Moreover, some basic but important information was lacking in the original study. At what age does the trait first appear? How long does it last? Is it sensitive to intervention with CGRP-related therapeutics etc?

Thus, we obtained a breeding pair of STA rats from the Thomas Jefferson University enabling us to generate successive generations of offspring for comparative validation purposes. Although not able to confirm the episodic fluctuating periorbital threshold as originally described by Oshinsky and colleagues^13^, we have observed that these rats exhibit a sex-independent, persistent cephalic hypersensitivity that emerges after puberty into adulthood which is highly sensitive to treatment with migraine-specific therapeutics.

## Materials and Methods

### Animals

All experiments were performed in STA rats derived from a single breeding pair originally sourced from the Thomas Jefferson University under licence^13^ and subsequently bred in house. Periorbital thresholds for this breeding pair, measured via manual von Frey filament testing^13^ are shown in Supplementary Table S1. For control purposes, SD rats were obtained from (Taconic Bioscience, Ejby, Denmark), Charles River (Sulzfeld, Germany) or were bred in house. The source, sex, age and body weight of rats used in each experiment is indicated in the study outline below. All rats were group housed in Tecniplast 1354G Eurostandard type IV polycarbonate cages (L*W*H: 60*38*20 cm; Brogaarden, Denmark) using a 12 hour light/dark cycle with lights on at 04.00. Individual opaque red polycarbonate shelters (20*11.5*16 and 15*9*9 cm respectively), together with an aspen biting stick (10*2*2 cm; Tapvei, Estonia) and piece of hemp rope suspended from the cage lid were provided in each homecage for retreat and enrichment purposes. Bedding consisted of Enviro-Dri nesting material (Brogaarden, Denmark). Standard rat chow (Altromin) and tap water were available ad libitum in the animals homecage environment. Humidity ranged from 45–65%. Experiments were approved by the Danish Animal Experiments Inspectorate and performed in accordance with the relevant guidelines and regulations of approval numbers 2012-15-2934-00697 and 2014-15-0201-00256.

### Study outline

In a first cohort of F1 (first generation) adult female STA rats (241–283 g), over a 12 day period, we evaluated the effects of cutaneous mechanical stimulation on periorbital and hindpaw thresholds and compared with control SD rats (230–362 g) sourced from Taconic or Charles River. The putative effect of oestrus cycle status on periorbital thresholds was also determined in these animals. We also assessed the general behavioural performance of these rats in LABORAS.

In a subsequent cohort of F3 (third generation) female (4–18 weeks old; 65–279 g) and male (11–15 weeks old; 367–516 g) STA rats we followed the effects of cutaneous mechanical stimulation on the development of periorbital thresholds prior to, and after puberty (e.g. in the same rats as juveniles, adolescents and adults), and compared with age-matched control female SD rats (91–388 g) and male SD rats (510–711 g) bred in our own animal facility; the parents of these rats were originally sourced from Charles River (Sulzfeld, Germany). Hindpaw thresholds to noxious mechanical and thermal stimulation, analgesic sensitivity to buprenorphine and general behavioural performance of the female rats in LABORAS was also evaluated. Finally, we assessed sensitivity to sumatriptan and olcegepant using a blinded cross-over paradigm in the same female STA and control SD rats.

In a final cohort of F4 (fourth generation) female (14 weeks old; 213–247 g) STA rats we compared the effects of cutaneous mechanical stimulation measured over different regions of the face and head with female control SD rats (21 weeks old; 332–420 g) bred in house. We also measured hindpaw thresholds in both groups of rats using two different assay methods as described below.

Where possible all behavioural analysis was performed with the investigator(s) blinded to rat substrain. All pharmacology experiments were performed with the investigator(s) blinded to treatment, with rats randomly allocated to vehicle or treatment groups.

### Measurement of periorbital thresholds after mechanical stimulation

The sensitivity of the frontal region of the head (V1 ophthalmic trigeminal dermatome), to static mechanical stimulation was measured using an electronic von Frey device fitted with a rigid plastic tip (IITC LifeScience Inc, USA). The rat was gently restrained using a cotton towel and held in a prone position in the lap of the investigator with its head and neck region left exposed and unrestricted. The tip of the device was then applied with increasing force (maximum of 450 g) to the right periorbital area above the eye, the midline and then the left periorbital area above the eye until the rat withdrew its head, laterally rotated its head and/or vocalized. The soft tissue around the eye was carefully avoided. The average of the three measurements was considered the withdrawal threshold (g). If a stimulus had to be reapplied due to inappropriate application care was taken not to apply the probe tip to the exact same location within the region being measured. The whole procedure typically took less than 2 minutes to perform.

The sensitivity of the V2 (maxillary) and V3 (mandibular) trigeminal dermatomes, and the cervical region of the head (between the ears) to mechanical stimulation was also measured in the cohort of F4 female STA and control SD rats. The same procedure as described above for the V1 (ophthalmic) region was applied for the cervical region with the average of the three measurements (right, midline and left) considered as the withdrawal threshold (g). However, for the V2 and V3 dermatomes only one measure per side was obtained; thus the average of two measurements was considered the withdrawal threshold (g) for these areas.

In order to determine if the method produced any lowering of the periorbital threshold between the first and last measure, or if any sided-ness occurred of relevance to the clinical manifestation of migraine^11^, we performed a crossover experiment over two sequential days, incorporating two testing sessions on each day (4 sessions in total) with the cohort of F3 female STA and control SD rats. Accordingly, in the first session the tip of the von Frey device was applied to the right periorbital area and then the left periorbital area in half of the rats of each substrain. The reverse process was followed in the remaining rats. The same process was then repeated in the second testing session. The following day, all rats were crossed over so that rats tested first over the right periorbital area on day one were tested first over the left periorbital area on day two and vice versa. Withdrawal threshold values (g) for each side were then averaged and compared for each substrain.

In light of retrospective findings showing that STA rats had smaller bodyweights than age-matched control SD rats, we reasoned that this might impact upon the assessment of periorbital thresholds to mechanical stimulation. Therefore, we decided to make a comparison of periorbital thresholds between STA and control SD rats over the same range of body weights (92–250 g). For individual rats, periorbital thresholds were plotted as a function of body weight on each day of testing within this range. We then performed a linear regression analysis of the individual data sets enabling a slope coefficient to be obtained for individual animals. The slope coefficients of all STA rats and control SD rats were then used for purposes of statistical comparison.

### Measurement of hindpaw thresholds after mechanical or thermal stimulation

Hindpaw sensitivity to mechanical stimulation was assessed using an electronic Randall Selitto paw pressure device (IITC LifeScience Inc, USA) as described previously^14^. Briefly, the rat was gently restrained within the hand and lower arm of the investigator and the right hindleg extended and the hindpaw inverted. The probe tip was then placed on the paw between the foot pads and increasing force uniformly applied (maximum of 450 g) until the rat withdrew the hindpaw and/or vocalized. The average of three measurements obtained from defined parts of the hindpaw excluding the footpads was considered the paw pressure withdrawal threshold (g). The exact same protocol was used to assess hindpaw sensitivity using the electronic von Frey device.

To assess hindpaw thresholds to noxious thermal stimulation we used a hot/cold plate (IITC) with a pre-set plate temperature of 48 °C. The latency (s) to respond to the thermal stimulus which consisted of either licking, rapid shaking, or stepping of the hindpaws, was recorded from the moment the rat was placed onto the surface of the plate. A cut-off-time was set at 60 seconds. No rats showed signs of thermally-induced damage to the paws throughout the duration of the study.

### Automated measurement of spontaneous behaviour using LABORAS

Analysis of time spent performing spontaneous behaviours (we chose to measure distance travelled and grooming) was evaluated in individual STA and control SD rats using an automated behavioural registration platform (LABORAS; Metris, Netherlands). This system consists of individual solid floor cages (8 in total in our set-up), covered with bedding material, which in turn are placed on platforms sensitive to vibrations caused by the animal. Different behaviours are associated with specific vibration patterns and identified by specific software algorithms^15^. Given the motor-based nature of the detection method we assessed the sensitivity of the system and the two behavioural endpoints in control SD rats sourced from Charles River to the GABA_A_ receptor positive allosteric modulator midazolam and the NMDA receptor blocker ketamine.

### Determination of oestrus cycle status

A sample for oestrous cycle determination was collected by vaginal lavage^16^ from F1 STA rats immediately after assessment of periorbital and/or hindpaw thresholds to mechanical stimulation. The samples were evaluated under a light microscope at 10X magnification (Nikon Eclipse *Ni*) and categorized into two categories due to the limited sample size; metoestrus/dioestrous and proestrous/oestrous.

### Drugs

The selective 5HT_1B/1D_ receptor agonist sumatriptan (GSK Denmark, Imigran 12 mg/ml; 1 mg/kg) was diluted in 0.9% saline and administered s.c. The CGRP receptor antagonist olcegepant (HCl salt; 1 mg/kg) was dissolved in 0.9% saline and administered i.p. The partial μ-opioid receptor agonist buprenorphine (Indivior UK, Temgesic 0.3 mg/ml; 0.1 mg/kg) was diluted in 0.9% saline and administered s.c. The GABA_A_ receptor positive allosteric modulator midazolam (Midazolam Hamelm Pharma Plus Germany, 1 mg/ml; 0.3 mg/kg) was diluted in 0.9% saline and administered i.p. The NMDA receptor antagonist ketamine (MSD Animal Health Ketaminol Vet. 100 mg/ml; 30 mg/kg) was diluted in 0.9% saline and administered i.p. All drugs were administered in a dosing volume of 1 ml/kg, and obtained from Glostrup Hospital Pharmacy except olcegepant HCl which was obtained from MedChemExpress (MedChemTronica AB, Sweden).

### Data handling and statistical analysis

Group sizes for the pharmacology experiments on cutaneous threshold values were estimated as a function of the desired effect size (approximately 50% change versus corresponding vehicle treatment or with control SD values), where we assumed a significance level of 5% and a power of 90%^17^. Statistical analysis was performed using GraphPad Prism 5 (Graph Pad Software Inc., San Diego, CA). For normally distributed data analysis of variance (ANOVA) was used to analyze the overall effects of treatments followed by Bonferroni’s multiple comparisons test. Direct comparison of treatments was made via paired or unpaired t tests as appropriate. For data not normally distributed around the mean or with unequal variances the specific tests used are mentioned as appropriate. All data are presented as mean ± S.E.M. P < 0.05 was considered to be statistically significant.

### Data availability

The datasets generated and analysed during the current study are available from the corresponding author on reasonable request.

## Results

### STA rats exhibit a selective cephalic hypersensitivity to mechanical stimulation

**Figure 1:**
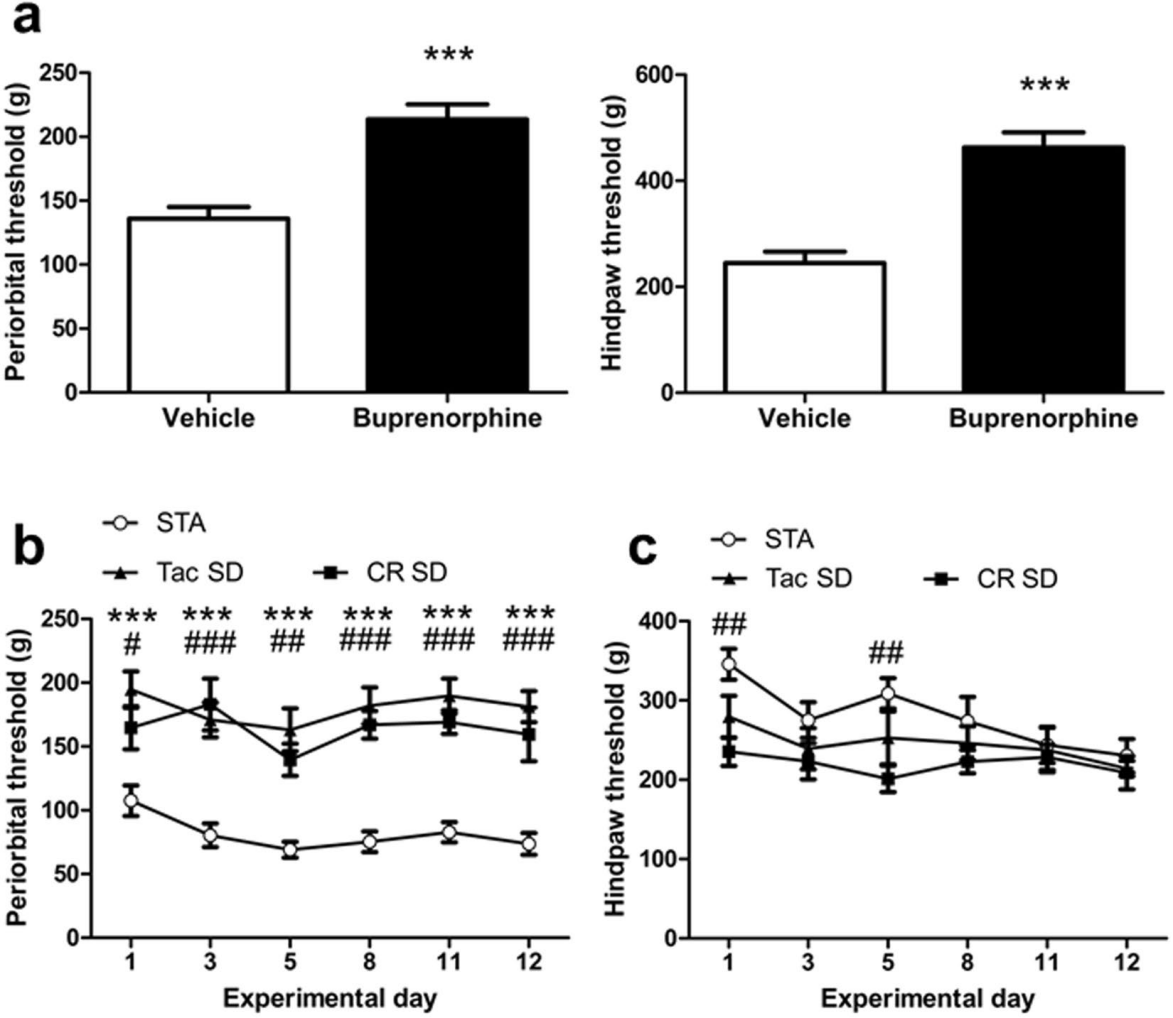
Selective presence of cephalic hypersensitivity in female STA rats. Hypersensitivity was assessed using an automated von Frey device and a paw pressure algesiometer to measure periorbital and hindpaw cutaneous thresholds (g) respectively. (**a**) Buprenorphine (0.1 mg/kg, s.c., n = 12) increased thresholds in adult female SD rats sourced from Taconic confirming assay sensitivity. (**b**) Periorbital thresholds in female F1 STA rats (n = 14) versus female SD rats sourced either Taconic (n = 13) or Charles River (n = 10). (**c**) Hindpaw thresholds in female F1 STA rats versus female SD rats sourced either Taconic or Charles River. Day number represents the day of testing. Data represent mean ± S.E.M. (**a**) ***P < 0.001 vs Vehicle unpaired t-test (**b**) and (**c**) ^#^P < 0.05, ^##^P < 0.01, ^###^P < 0.001 vs SD CR; ***P < 0.001 vs SD Tac; two way RM ANOVA followed by Bonferonni’s.

The presence of a selective cutaneous mechanical hypersensitivity in STA rats has thus far only been described in one study^13^. Prior to assessing periorbital and hindpaw thresholds in STA rats using the electronic von Frey device and Randall-Selitto paw pressure algesiometer, measures of assay sensitivity were obtained by assessing the analgesic effects of buprenorphine (0.1 mg/kg, s.c.) in naïve adult female SD rats. As expected, buprenorphine treatment significantly increased periorbital, and hindpaw thresholds compared with vehicle t(22) = 5.166, P < 0.001 and t(22) = 6.139, P < 0.001 respectively, unpaired t test (Fig. 1a).

Having validated the sensitivity of the methodology, we proceeded to measure periorbital and hindpaw thresholds in a cohort of F1 adult female STA rats over a 12 day period, and compared with adult female SD rats sourced from either Taconic or Charles River. STA rats had a two fold lower periorbital threshold to mechanical stimulation compared with the other two SD substrains and this effect was maintained consistently over time F(2,34) = 37.92, P < 0.001, (Fig. 1b). In contrast, no consistent difference in hindpaw thresholds to mechanical stimulation was observed between STA rats and Taconic or Charles River SD rats (Fig. 1c). While this data generally supports the observations of Oshinsky *et al*.^13^ they do not recapitulate them entirely e.g. individual STA rats did not present with episodic fluctuating periorbital thresholds. No difference (t(15) = 0.1617, P = 0.874, paired t-test) in periorbital thresholds was observed between STA rats during Metoestrus/Dioestrus versus Prooestrus/Oestrus (Supplementary Figure S1).

**Figure 2:**
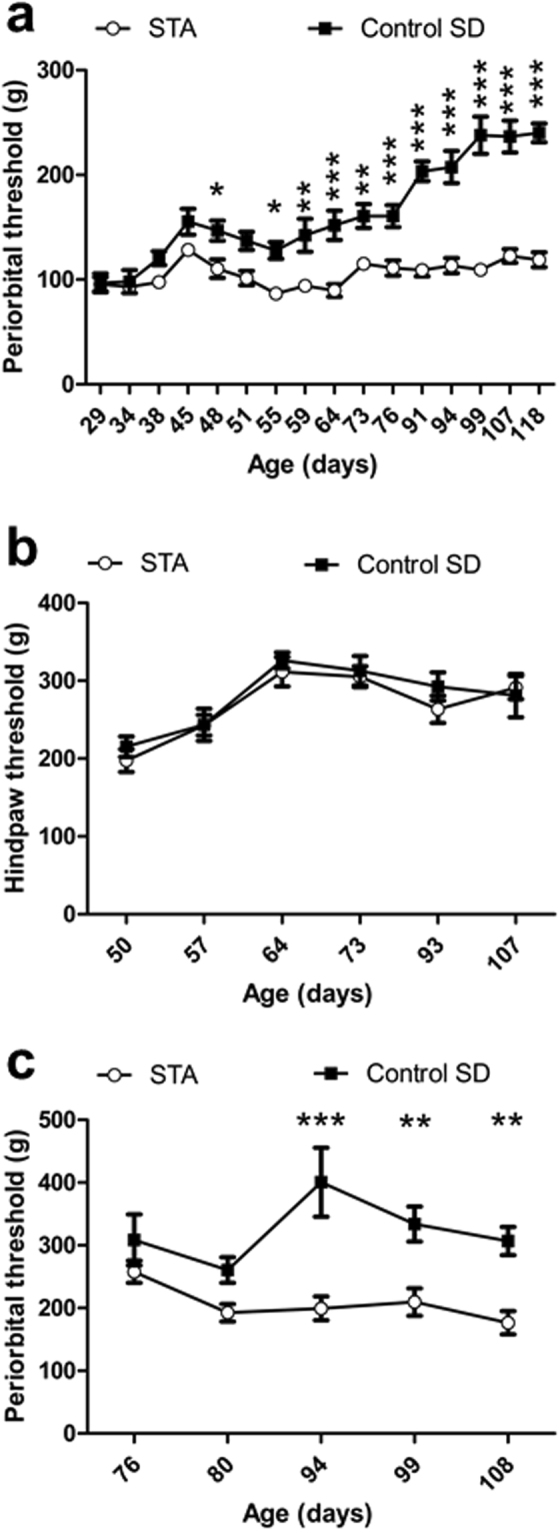
Development of cephalic hypersensitivity occurs after puberty in STA rats. Hypersensitivity was assessed in F3 STA rats and age-matched control SD rats bred in house during different periods encompassing pre- and post-puberty. An automated von Frey device and paw pressure algesiometer were used to measure cephalic and hindpaw sensitivity to mechanical stimulation respectively. (**a**) Periorbital thresholds in female STA rats (n = 17) and control SD rats (n = 8) (**b**) Hindpaw thresholds in female STA rats (**c**) Periorbital thresholds in male STA rats (n = 8) and control SD rats (n = 4). Data represent mean ± S.E.M. *P < 0.05, **P < 0.01, ***P < 0.001 vs control SD; two way RM ANOVA followed by Bonferonni’s.

Next, we measured the periorbital and hindpaw thresholds of a separate cohort of F3 STA rats over a much longer time period encompassing puberty, through adolescence into early adulthood (aged 29–118 days)^18^. We also wanted to exclude the possibility that the observed phenotype of STA rats might simply occur as a consequence of environmental influences experienced by the different rat substrains during the pre- and/or post-natal periods. Thus, we compared periorbital and hindpaw thresholds of F3 female STA rats with female SD rats bred in house, the parents of which were sourced from Charles River. After weaning at 4 weeks, comparison of periorbital thresholds to mechanical stimulation revealed a significant difference between the substrains (F(1,23) = 130.3, P < 0.001) as shown in Fig. 2a. Again, we found no difference in hindpaw thresholds to mechanical stimulation between STA rats and corresponding SD controls (Fig. 2b). Although not the primary focus of our attention in this cohort of F3 rats, we also confirmed that STA males exhibited a persistent hypersensitivity to periorbital mechanical stimulation (F(1,10) = 19.08, P = 0.001), (Fig. 2c). We did not exclude any STA rats from our data analysis as a consequence of not displaying low periorbital thresholds to mechanical stimulation. Thus, the overall frequency of cutaneous cephalic hypersensitivity (100%) was identical between female and male STA rats.

We also assessed mechanical thresholds distributed over the face and towards the back of the head in a final cohort of F4 female STA rats to determine if the cutaneous hypersensitivity measured over the periorbital region within the trigeminal V1 dermatome was specific to this area. Table 1 shows that these STA rats also had significantly lower thresholds to mechanical stimulation within the trigeminal V2 (maxillary) and V3 (mandibular) dermatomes, and the cervical region compared with control SD rats (P = 0.01, P < 0.001 and P < 0.001 respectively, unpaired t test). Finally, in the same F4 STA and control SD rats, measurement of hindpaw mechanical thresholds using either the electronic Randall-Selitto paw pressure algesiometer or the electronic von Frey device revealed that the two methods provided slightly different absolute threshold values (Table 1). Importantly, both methods revealed that hindpaw threshold values were not significantly different between STA and control SD rats.

**Table 1:** Comparison of cephalic, facial and hindpaw cutaneous sensory thresholds to mechanical stimulation in STA rats.

	Threshold (g)					
	Periorbital V1	Maxillary V2	Mandibular V3	Cervical	Hindpaw eVF	Hindpaw eRS
STA	87.1 ± 13.3	86.7 ± 11.9	98.1 ± 5.5	102.0 ± 3.0	195.9 ± 5.9	241.6 ± 10.0
control SD	167.4 ± 8.3	124.2 ± 4.0	163.7 ± 9.5	158.4 ± 7.3	183.7 ± 4.7	243.7 ± 20.6
P value	<0.001	0.01	<0.001	<0.001	0.114	0.921

Thresholds over the V1, V2 and V3 trigeminal dermatomes (incorporating the periorbital, maxillary and mandibular regions respectively), and the cervical region to mechanical stimulation were assessed in F4 female STA (n = 8) and control SD rats (n = 7) using an electronic von Frey device. For comparison hindpaw thresholds to mechanical stimulation were assessed using an electronic von Frey (eVF) and an electronic Randall-Selitto (eRS) paw pressure algesiometer in the same animals. Data represent mean ± S.E.M. P value represents STA vs control SD, unpaired t-test.

### STA rats display a paradoxical hindpaw hypoesthesia to thermal stimulation

**Figure 3:**
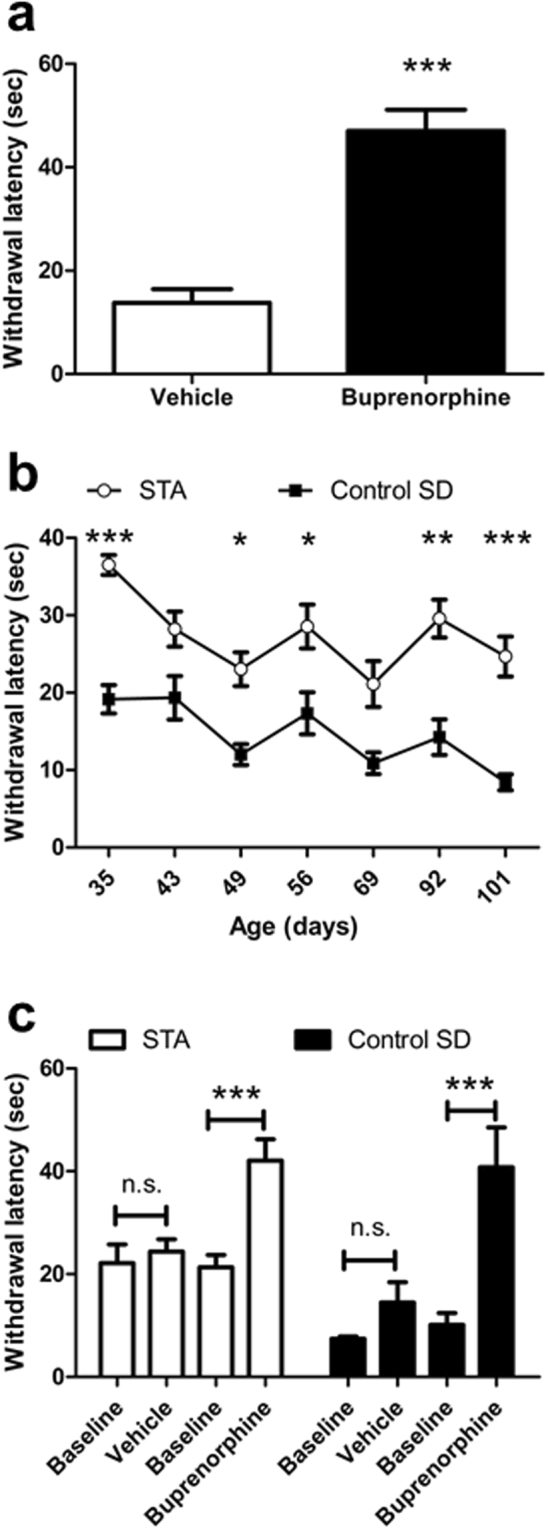
STA rats display a paradoxical hindpaw hypoesthesia to noxious thermal stimulation. The latency to hindpaw withdrawal (s) in response to a 48 °C noxious thermal stimulus was assessed using the hot plate test. (**a**) Buprenorphine (0.1 mg/kg, s.c., n = 12) increased withdrawal latency in adult female SD rats sourced from Taconic confirming assay sensitivity. (**b**) Withdrawal latency in female F3 STA rats (n = 16) versus age-matched female SD rats (n = 7) bred in house during different periods encompassing pre- and post-puberty (**c**) Buprenorhine (0.1 mg/kg, s.c.) mediated a greater degree of analgesia in female F3 STA (n = 12) rats versus female SD rats (n = 7) bred in house. Data represent mean ± S.E.M. (**a**) ***P < 0.001 vs Veh, unpaired t test (**b**) *P < 0.05, **P < 0.01, ***P < 0.001 vs control SD, two way RM ANOVA followed by Bonferonni’s (**c**) ***P < 0.001 vs corresponding baseline, one way ANOVA followed by Bonferonni’s.

Peripheral sensory afferents that encode noxious information respond to a range of stimuli including mechanical, heat, cold and chemical provocation^19^. To ensure that the lack of apparent hindpaw sensitivity to mechanical stimulation of these STA rats was not simply due to the type of stimulus applied we also assessed their response to noxious thermal stimulation using the hot plate test. Firstly, we obtained a comparative measure of assay sensitivity by assessing analgesic effects of buprenorphine in adult female SD rats sourced from Taconic. As expected, buprenorphine treatment significantly increased the latency to respond compared with vehicle (t(22) = 6.812, P < 0.001), (Fig. 3a). Surprisingly, F3 female STA rats were shown to exhibit almost a two fold increase in the latency to respond to noxious thermal stimulation of the hindpaw compared with their control SD rats at the first time of testing on Day 35. Moreover, Fig. 3b illustrates that this hypoesthesia was consistently maintained over time (F(1,21) = 40.21, P < 0.001).

To better understand a possible mechanistic basis for the observed thermal hypoesthesia we assessed the putative sensitivity of endogenous pain pathways by challenging the same F3 female STA and control SD rats with buprenorphine. As expected, a significant effect of buprenorphine (0.1 mg/kg, s.c.) treatment was obtained in control SD rats (F(3,6) = 13.79, P < 0.001). Buprenorphine also produced a clear analgesia in STA rats (F(3,11) = 9.315, P < 0.001). However, compared with corresponding vehicle treatment the increase in withdrawal latency after buprenorphine was 2 fold less in STA rats versus control SD rats (Fig. 3c), indicating the possible presence of analgesic tolerance to buprenorphine, and commensurate with a persistent recruitment of opioid-sensitive inhibitory pain pathways relevant to thermal nociceptive processing.

### Validation of automated von Frey assay and general behavioral phenotype of STA rats

**Figure 4:**
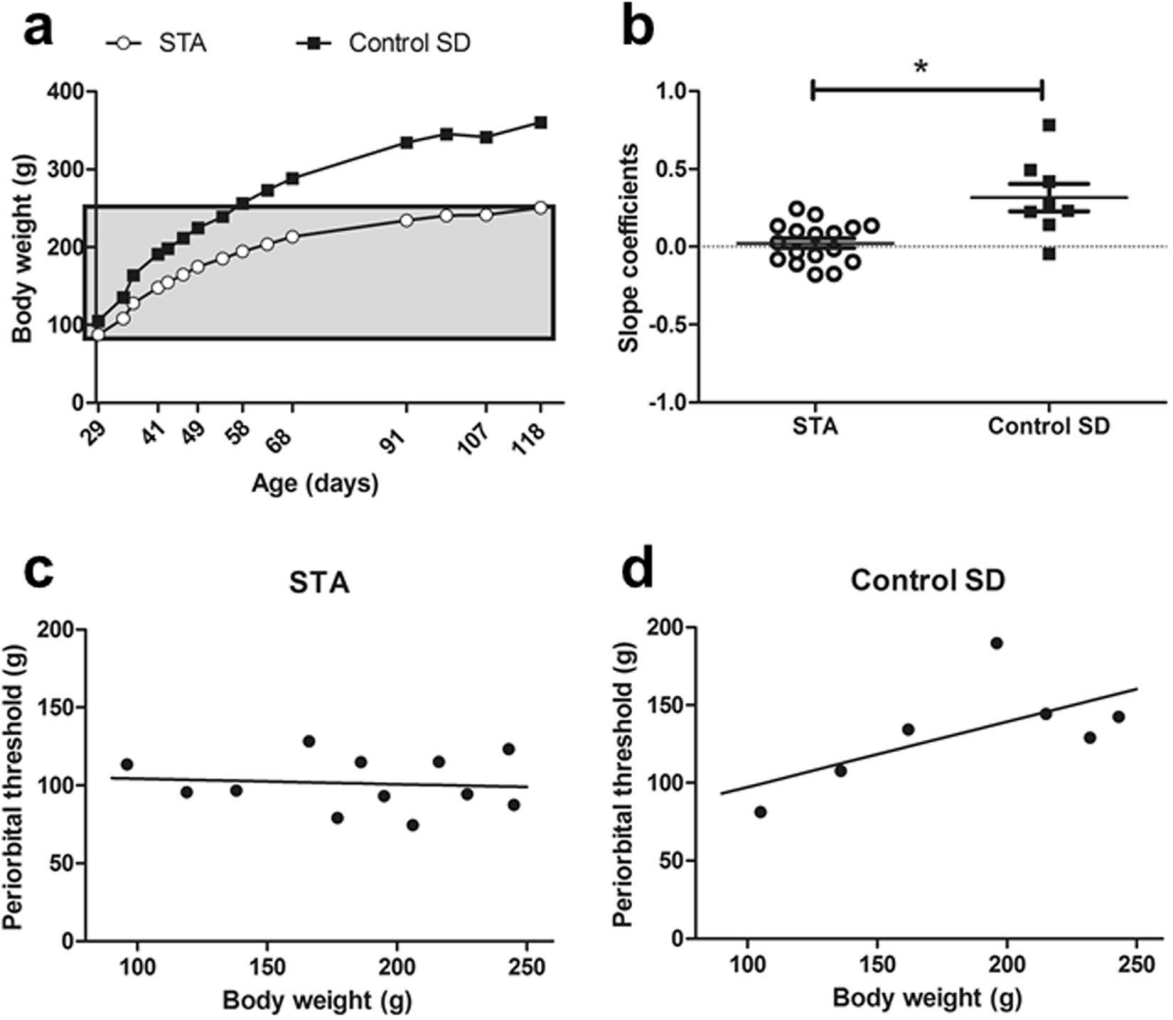
Body weight gain and correlation to periorbital thresholds in female STA rats. (**a**) Body weight changes over time in F3 female STA rats (n = 17) compared with age-matched female control SD rats (n = 8) bred in house. The shaded area indicates the body weight range (92–250 g) that was subsequently used to enable slope coefficients for each substrain to be determined. (**b**) Comparison of periorbital thresholds with body weight enabled slope coefficients for individual rats to be calculated. These were then grouped and compared to reveal differences in coefficients between the sub-strains indicating that the lower periorbital thresholds consistently observed in STA rats align to a specific cephalic hypersensitivity. (**c,d**) representative examples of body weight correlated to periorbital threshold over the range of 92–250 g for individual STA and control SD rats showing slope coefficients. Data represent mean ± S.E.M. *P < 0.05 vs Control SD, unpaired t test.

In the three cohorts of STA rats included here their general health and welfare status appeared normal. They showed no signs of skin or coat problems, displayed normal posture when sitting, standing and/or rearing (e.g. no hunching or belly dragging) and had normal mobility (e.g. no ataxia or limb splay). STA rats also appeared to interact normally with each other. However, Fig. 4a shows that F3 female STA rats weighed less upon weaning and gained weight less rapidly compared with control SD rats bred in house. A consequence of the attenuated weight gain in STA rats could be that this affects their ability to respond robustly to periorbital stimulation as they age. Notably, a positive correlation between body weight and periorbital thresholds was occasionally seen in both STA and control SD rats (Figs 2a and 4a). However, regressional analysis revealed that the slope of coefficient was significantly different between STA and their SD controls t(8) = 3.131, P < 0.014, unpaired t test with Welch’s correction; (Fig. 4b–d). Thus, we are confident that following puberty STA rats possess a specific cephalic hypersensitivity *per se*.

We also applied the von Frey mechanical stimulus in the reverse order across the periorbital regions (e.g. left to right) in female F3 STA rats to assess the possibility that (i) the hypersensitivity might preferentially be unilateral in its distribution^11^ (ii) threshold measurements might decrease with repeated testing. Notably, we were unable to detect any effect of unilaterality or influence of repeated application of mechanical stimulation on periorbital thresholds (Supplementary Figure S2).

**Figure 5:**
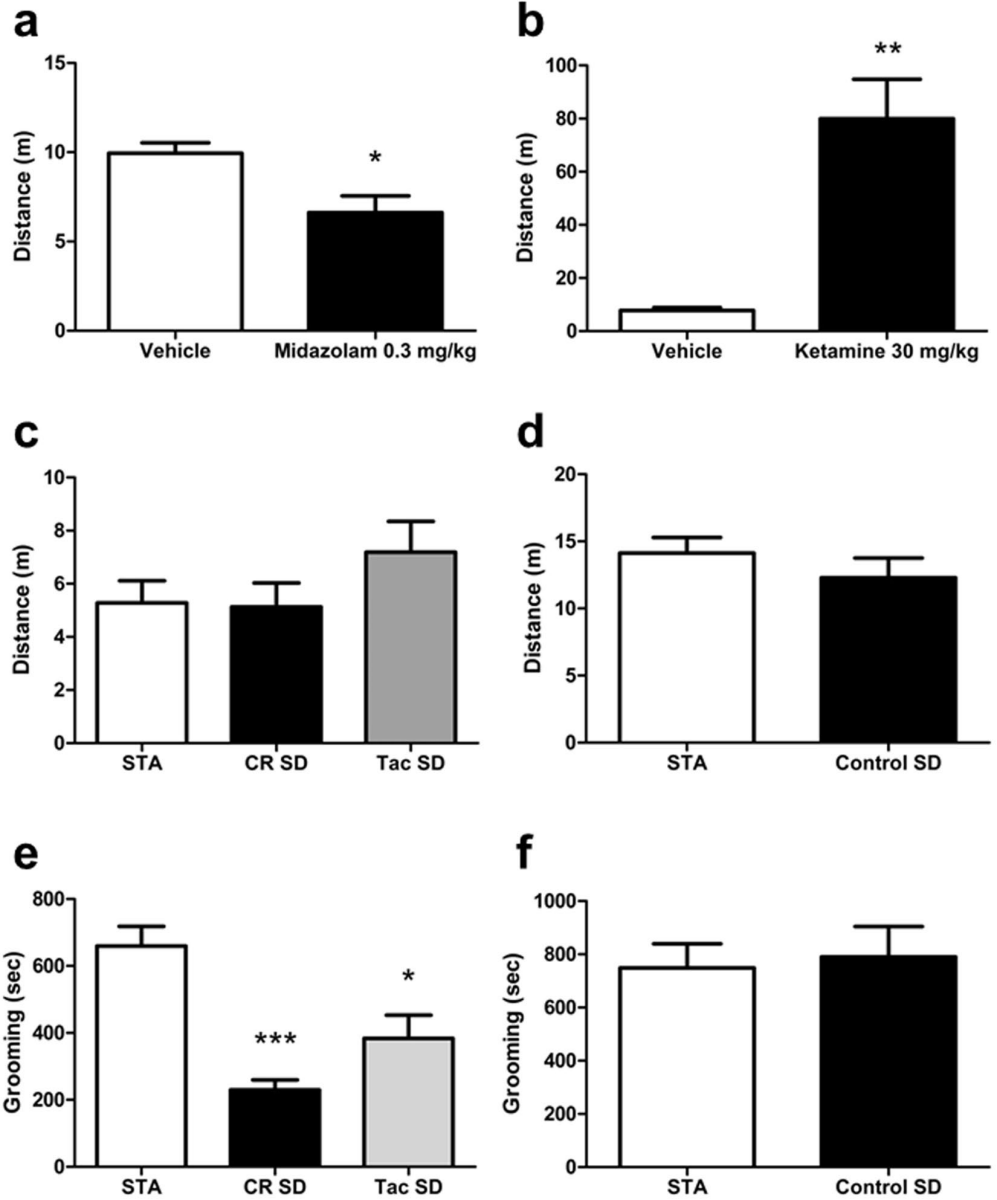
STA rats do not exhibit clear deficits in general behaviour. Spontaneous behaviour was measured via automated profiling of discrete motor-related body movements using LABORAS. Effect of (**a**) midazolam (0.3 mg/kg, i.p., pre-treatment time = 30 mins, n = 6), Vehicle (n = 6) and (**b**) ketamine (30 mg/kg, i.p., pre-treatment time = 30 mins, n = 9), Vehicle (n = 9) on distance travelled (m) in control SD rats sourced from Taconic as an index of assay sensitivity. No change in distance travelled in (**c**) STA female F1 STA (n = 14) rats versus female SD rats sourced either Charles River (n = 10) or Taconic (n = 13) and (**d**) female F3 STA rats (n = 16) versus age-matched female control SD rats (n = 8) bred in house. (**e**) Time spent grooming (secs) in female F1 STA rats versus female SD rats sourced either Taconic or Charles River. (**f**) Time spent grooming (secs) in female F3 STA rats versus age-matched female control SD rats bred in house. Note that the total time spent grooming by STA rats was similar in (**e**) and (**f**). Data represent mean ± S.E.M. (**a**) *P < 0.05 vs Vehicle, unpaired t test, (**b**) **P < 0.01 vs Vehicle unpaired t test with Welch’s correction (**e**) *P < 0.05, ***P < 0.001 vs Vehicle, Kruskal-Wallis followed by Dunn’s.

To further evaluate the general behavioural phenotype of STA rats we analysed a range of spontaneous behaviours using the automated behavioural registration system LABORAS. The sensitivity of the system to detecting both decreases and increases in motor-based behaviours was confirmed in naïve SD rats administered midazolam t(10) = 3.003, P = 0.013, unpaired t test and ketamine t(8) = 4.842, P = 0.001, unpaired t test with Welch’s correction respectively, (Fig. 5a and b). Subsequently, comparison of either F1 or F3 female rats with corresponding control SD rats revealed no difference in distance travelled between any of the substrains (F(2,34) = 1.366, P = 0.269 and t(22) = 0.936, P = 0.359, unpaired t-test) as shown in Fig. 5c and d. Finally, whereas F1 female STA rats appeared to groom more versus vendor sourced SDs (H(2) = 19.05, P < 0.001 Kruskal-Wallis; Fig. 5e), comparison of F3 female STA rats with in house bred control SD rats revealed no change in grooming behaviour (t(22) = 0.274, P = 0.787 Fig. 5f).

### Pharmacology of cephalic threshold responses in STA rats

**Figure 6:**
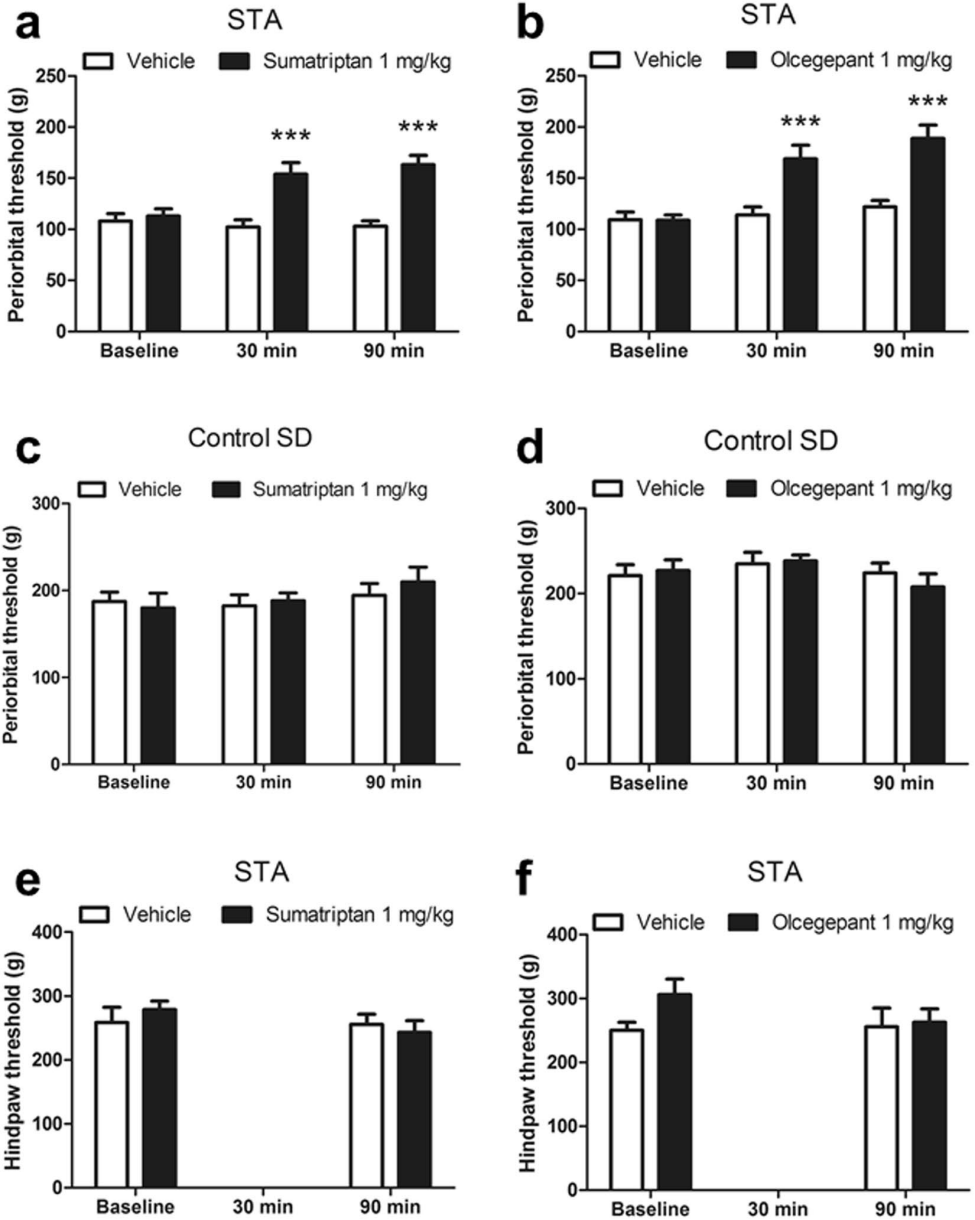
Reversal of cephalic hypersensitivity in STA rats with sumatriptan and olcegepant. Adult female F3 STA rats with established cephalic hypersensitivity were administered an acute systemic injection of drug or vehicle 1 hour after baseline cutaneous periorbital thresholds (g) to mechanical stimulation had been measured. Effect of **(a**) sumatriptan (1 mg/kg, s.c., n = 13) or Vehicle (n = 13) (**b**) olecegepant (1 mg/kg, i.p., n = 13) or Vehicle (n = 13). Periorbital thresholds were then measured again at 30 and 90 mins post-injection. To confirm the selectivity of the sumatriptan and olecegepant responses, effects on periorbital thresholds were also measured in (**c,d**) age-matched female control SD rats (all groups n = 8) bred in house and (**e,f**) hindpaw thresholds (g) in the same STA rats measured using the paw pressure test (all groups n = 13). A within subjects crossover treatment protocol was used with two days washout between treatments and 1 week washout between sumatriptan and olcegepant experiments. Data represent mean ± S.E.M. ***P < 0.001 vs Vehicle, one way RM ANOVA followed by Bonferonni’s.

Confident that successive generations of STA rats exhibited a sustained cephalic hypersensitivity compared with control animals, we next set out to assess efficacy of migraine-relevant therapeutics in STA rats. Accordingly, acute systemic administration of sumatriptan (1 mg/kg, s.c.) to adult female F3 STA rats significantly elevated periorbital thresholds compared with vehicle treatment at 30 and 90 mins (treatment × time interaction; F(2,24) = 7.571, P = 0.001) as shown in Fig. 6a. Moreover, comparison of the post treatment sumatriptan response for each animal with its corresponding baseline response also revealed a significant effect of sumatriptan treatment F(1,24) = 28.78, P < 0.001. In contrast, sumatriptan had no effect on periorbital thresholds in control SD rats or on hindpaw thresholds in STA rats (Fig. 6c and e).

Figure 6b shows that acute systemic administration of olcegepant (1 mg/kg, i.p.) to adult female STA rats also significantly elevated periorbital thresholds compared with vehicle treatment at 30 and 90 mins (treatment × time interaction; F(2,24) = 11.98, P < 0.001. Comparison of the post treatment olcegepant response for each animal with its corresponding baseline response also revealed a significant effect of olcegepant treatment F(1,24) = 15.33, P < 0.001. As was the case with sumatriptan, olcegepant had no effect on periorbital thresholds in control SD rats or on hindpaw thresholds in STA rats (Fig. 6d and f). Accordingly, the efficacy data obtained with two distinct clinically relevant migraine therapeutics in STA rats strongly support that these drugs targeted migraine-relevant pathophysiological mechanisms engaged within trigeminal pain circuits.

## Discussion

STA responding rats have been reported to display signs of migraine-related behaviour consisting of episodic fluctuating periorbital cutaneous hypersensitivity to mechanical stimulation with von Frey monofilaments^13^. Originally derived from the Sprague-Dawley strain obtained from Charles River, 50–75% penetrance of the trait occurred by the tenth generation of offspring. Upon receipt of a breeding couple (F19) derived from this colony of rats we subsequently bred four further generations of STA rats, and confirmed that both male and female post-pubescent STA rats exhibit a generalized cephalic hypersensitivity that becomes more robust through adolescence into adulthood. Crucially, we were able to show that the hypersensitivity is sensitive to treatment with migraine-specific drugs.

Migraine attacks can be associated with symptoms of cutaneous allodynia^20,21^, which occur more frequently in chronic migraineurs^22^. We exploited this feature to show that different generations of STA rats present with consistently lower periorbital thresholds to mechanical stimulation when compared with SD rats obtained from either external vendors or when bred in house. Intriguingly, this sensitization was not limited to just the trigeminal V1 dermatome, since we also observed that STA rats were more sensitive to mechanical stimulation applied over the trigeminal V2 and V3 dermatomes, and over the cervical region innervated by the greater occipital nerve. Since the epidemiological prevalence of migraine in the general population is greater in female than male subjects^23^, we focused the majority of our attention to using female STA and SD control rats for experimental purposes.

Migraine prevalence increases dramatically during and after puberty^24^ and peaks in both sexes during adulthood from 25–55 years of age^25^. Having initially identified a migraine-relevant behavioural disturbance in a cohort of F1 adult STA rats, we were curious to know at what developmental stage it emerged. Accordingly, we confirmed that cephalic sensitivity first became apparent in F3 female STA rats at around 48 days of age, a point at which *Rattus Norvegicus* is generally acknowledged to have undergone puberty^18^. Thereafter, periorbital thresholds remained essentially unchanged throughout the duration of each STA rat’s lifetime. Notably, body weight gain was greater in control SD rats following puberty, raising the possibility that the difference in periorbital thresholds between strains might simply reflect this aspect of phenotype. Importantly, when we performed regressional analysis and compared the slope coefficients for periorbital threshold versus body weight we found a significant difference between F3 female STA rats and corresponding control SD rats. Thus, we are confident that STA rats display an apparent cephalic hypersensitivity in the weeks and months following puberty. Our data did not reveal a fluctuating or episodic periorbital hypersensitivity in STA rats of either sex in any of the generations tested in contrast to Oshinsky *et al*.^13^, and the original breeding pair as shown in Supplementary Table S1. Rather, we observed a low periorbital threshold measurement in each and every STA rat of both sexes possibly reflective of migraine chronification. Chronic migraine affects between 1–2% of the general population and can progress from episodic migraine in association with a number of risk factors including medication overuse, obesity, depression and stress. However, at present, specific pathophysiological mechanisms underpinning chronic migraine are not particularly well understood^26^.

For pragmatic purposes in our experiments, we applied a static mechanical stimulus with increasing force across the left, mid and right periorbital cutaneous regions of the rats using an electronic von Frey device^14^. By performing a carefully conducted blinded cross-over experiment as described in Supplementary Figure S1, we did not reveal any unilaterality, or effect of repeated stimulus application. Accordingly, we would describe the pressure sensitivity as bilateral in location, a feature of migraine headache that is not uncommon in migraine patients^27^. Typically, researchers including Oshinsky and coworkers^13^, use von Frey monofilaments to assess cutaneous mechanical hypersensitivity within the trigeminal nerve branches, including the periorbital area. Whilst both methods apply a static blunt force the latter procedure has the possible advantage in that it can performed in freely-moving animals. Our method transiently restricted the movement of the animal, albeit the nature of the applied restraint was nowhere near as severe as that used to induce a pathophysiological stress response in rodents^28^. Moreover, we routinely habituated all rats to the handling associated with this procedure in our experiments. In its favour, the electronic von Frey device applies a non-interrupted increasing force until the rat either moves its head - typically by making a lateral rotation - and/or vocalizes at which point the mechanical threshold is precisely obtained. In contrast, individual von Frey monofilaments are designed to produce only one single force at buckling point. Thus, a series of filaments is standardly used with the increasing forces applied in discrete non-linear steps and an estimation of the mechanical threshold obtained^29^. Caveats asides, we have recently shown that both methods are capable of detecting a cephalic hypersensitivity after acute administration of GTN in naïve rats^30^. In support of our current findings, a trend towards a more persistent hypersensitivity as opposed to fluctuating hypersensitivity in later generations of STA rats was apparently observed by Oshinsky and colleagues^13^.

Extracephalic cutaneous hypersensitivity can occur in migraine patients, although it is not as commonly reported as cephalic hypersensitivity^31^. In agreement with previous observations^13^, we saw no clear sensitivity to cutaneous mechanical stimulation of the hindpaw in STA rats. Importantly, the application of paw pressure with the automated algesiometer we used is sensitive enough to detect both a lowering of the mechanical threshold^14^ and an increase as shown here by the robust buprenorphine-mediated analgesia in control SD rats. Furthermore, we observed a similar outcome using the electronic von Frey device as shown in Table 1. From this, we would conclude that STA rats appear to possess a selective cephalic sensitivity to cutaneous mechanical stimulation which does not appear to extend beyond the trigeminal and cervical receptive field areas. This contrasts with animal migraine models induced by systemic administration of NO donors such as GTN or isosorbide dinitrate^1,2,10^, or even dural administration of algogenic mediators^3–5^, where depending on the dosing paradigm used a hindpaw hyperalgesia occurs.

An unexpected finding in the current study was the presence of a robust and persistent hindpaw hypoesthesia to noxious thermal stimulation in female STA rats. Moreover, this hypoesthesia was apparent both pre- and post-puberty in contrast to the putative gain of function reflected in low periorbital thresholds which only manifested after puberty. In a subsequent hot plate experiment, we noted that the analgesic efficacy of buprenorphine was diminished in STA compared with control SD rats, reflective of analgesic tolerance^32^. These findings indicate that opioid-mediated signaling events occurring within somatic pain circuits relevant to thermal nociceptive processing appear to be tonically activated in STA rats. We have not yet assessed thermal sensitivity over the periorbital area due to a lack of suitable methodologies available for this specific purpose in rodents. However, we are currently working to address this limitation as it raises a critical issue in relation to preclinical model development that is not trivial. Typically, when characterizing behaviours in a new animal disease model researchers focus on signs and symptoms relevant to pathophysiology of interest. The presence of other behavioural disturbances might not be reported. Thus, we chose to highlight the hypoesthesia described above. Likewise, we had already noted that STA rats appeared to gain weight less rapidly than control SD rats throughout their respective lifetimes. To address if this potential confound impacted on other aspects of behaviour we measured the presence of spontaneous behaviours in STA rats using LABORAS and compared with control SD rats^15^. Our initial data appeared to reveal that STA rats groomed more than outsourced SD control rats, which could possibly indicate a facet of facial or cephalic hypersensitivity since rodent models of migraine-like pain can involve focused attention to these regions. However, our data are ambiguous since comparison of F3 female STA rats with control female SD rats bred in house failed to reveal any differences in general behaviour between the two substrains. We plan to address this issue in future experiments by utilizing additional migraine-associated triggers such as food/water deprivation, sleep deprivation or a homotypic stressor such as restraint to improve assay sensitivity.

We were able to correlate the sustained reduction in periorbital thresholds observed in female STA rats with pharmacological sensitivity to acute administration of the migraine-relevant therapeutics sumatriptan and olcegepant. Notably, we saw no effect of sumatriptan on hindpaw thresholds in STA rats; although sumatriptan has been reported to reverse hindpaw inflammatory hyperalgesia in mice, this was seen only after intrathecal administration^33^. In addition, we saw no effect of sumatriptan on periorbital thresholds recorded from age-matched control SD rats. Thus, we are confident that sumatriptan targeted cephalic sensory neurones in accordance with its ascribed function in complimentary models of chemical and electrically-provoked trigeminal sensitization^2,10,34^ and its effectiveness as a clinically used migraine therapeutic. Our data also reveal that facilitated CGRP release and/or enhanced functioning of CGRP receptors contribute to pathophysiology in this model. This compliments the observation that CGRP exacerbates cephalic sensitivity in STA rats^13^.

An important aspect of the blinded cross-over experimental design we used here is that we could compare drug effects with respect to vehicle over time, and to within animal baseline responses. Crucially, comparison of inter-session efficacy for both sumatriptan and olcegepant after wash-out revealed a highly similar magnitude of threshold reversal between each session. We believe the exquisite reproducibility of the data reflects an established and highly conserved disease pathophysiology between STA rats. Moreover, the implementation of such a paradigm more closely reproduces standard practices used in the clinical setting. For pain and migraine alike, a lack of data reproducibility obtained in drug efficacy studies by independent research laboratories is just one, amongst a myriad of factors which could be deemed responsible for the lack of successful analgesic drug development over the past two decades^14^. Within pain research the strain of rat used, and even vendor supplying the same strain of rat has been shown to profoundly affect both behavioural outcome (as illustrated by our LABORAS findings), and the associated pharmacological sensitivity to standard of care analgesics after injury^35^. Typically, outbred strains such as SD or Wistar rats are used, presumably based on appreciable merits such as reproductive fecundity, ease of animal handling and adherence of researchers to previously published methodologies^36^. Whether there also exists a misguided assumption that the heterogenic diversity of such outbred strains better reflects that of a typical pain or migraine patient cohort is debatable.

At present, the utility of the STA model to identify novel, high value migraine targets compared with other more established preclinical migraine approaches involving e.g. chemical provocation in awake rats, or dural electrical stimulation in anesthetized rats for purposes of translationally-relevant drug development remains unexplored. In future experiments we plan to test standard preventative treatments as typified by topiramate, which has been shown to be effective in the treatment of chronic migraine^37^, in an attempt to further reconcile translational facets of STA rats as a model of chronic migraine-like pain. Moreover, the pathophysiological and genetic underpinnings responsible for the persistent functional phenotype of STA rats we have described here have yet to be unraveled. We believe we have made important progress towards providing an independent characterization of an inbred rat strain, wherein rats that should be isogenetically similar, are shown to exhibit a long-term stable cephalic hypersensitivity sensitive to migraine-specific therapeutics.

## Electronic supplementary material

Supplementary information
