# Supplementary material for: A unique inbred rat strain with sustained cephalic hypersensitivity as a model of chronic migraine-like pain
Source: Sci Rep. 2018 Jan 30;8:1836. doi: 10.1038/s41598-018-19901-1 (PMC5789845; doi:10.1038/s41598-018-19901-1)
Supplement: Supplementary file 1 — Supplementary information [file 41598_2018_19901_MOESM1_ESM.pdf]

A unique inbred rat strain with sustained cephalic hypersensitivity as a model of chronic migraine-like pain

Gordon Munro[1,*], Steffen Petersen[1], Inger Jansen-Olesen[1], Jes Olesen[1]

[1]Danish Headache Center, Department of Neurology, Glostrup Research Institute, Nordre Ringvej 69, 2600 Glostrup, Denmark
*gordon.munro@regionh.dk

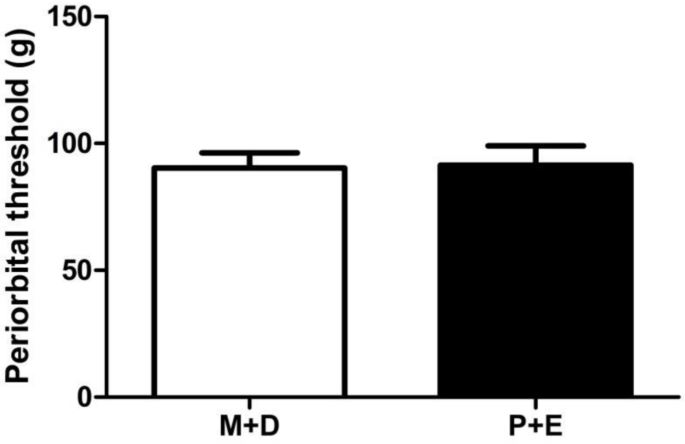

Supplementary Figure S1 – Cephalic hypersensitivity remains unchanged in female STA rats throughout the oestrus cycle. Periorbital thresholds (g) were measured in female STA rats (n=16) using an automated von Frey device. The four stages of the oestrus cycle (Metoestrus, Dioestrus, Prooestrus and Oestrus) were collapsed into (M + D) and (P + O) due to limited sample sizes within the groups. Oestrogen concentration in the plasma is highest during Prooestrus, whereas progesterone concentrations spike towards the end of this phase and begin to decline during Oestrus (1). Accordingly, comparison of the combined groups shown still provides important insight into putative effects of steroid hormones on cephalic hypersensitivity in STA rats. Data represent mean ± S.E.M.

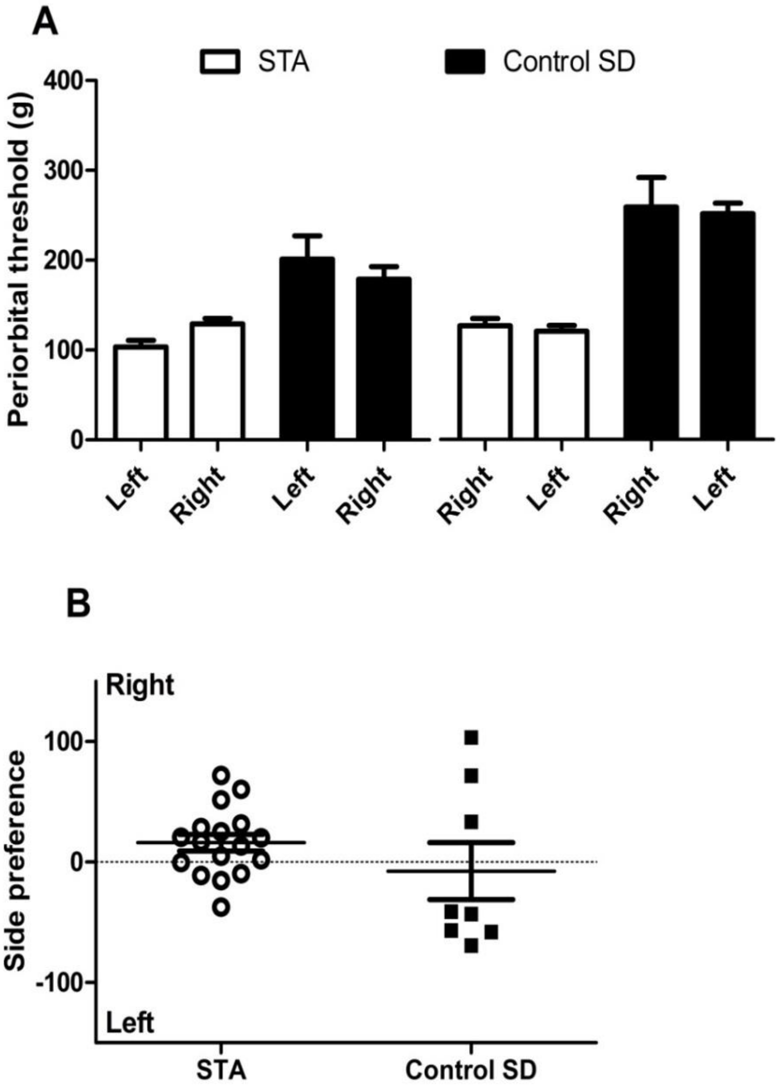

Supplementary Figure S2 – Mechanical testing does not sensitize STA rats and confirms the presence of a bilateral cutaneous cephalic hypersensitivity. Periorbital thresholds (g) were measured in F3 female STA (n=17) and age-matched control SD (n=8) rats using an automated von Frey device. A blinded cross-over design was used so that half the rats were tested first on the left side and then the right side with the process reversed for the remaining rats. The following day the process was repeated in reverse order. (**A**) No difference in periorbital thresholds was observed between the first and second measure in either substrain indicating that repeated testing did not sensitize the rats to cutaneous mechanical stimulation. (**B**) For each rat, the left and right periorbital thresholds (g) obtained in (**A**) were subtracted from each other to obtain an index of side preference. Negative and positive values indicate preference of the cephalic sensitivity to either the left or right side within individual rats. The similarity in distribution around the mean between STA and control SD rats indicates that the cephalic hypersensitivity was bilateral in nature. Data represent mean ± S.E.M.

Rat ID	Sex	Date of birth	Day 1	Day 2	Day 3	Day 4	Day 5	Day 6	Day 7	Day 8	Day 9	Day 10	Day 11	Day 12	Day 13	Day 14
F18-1-2	F	01-09-14	2	2	4	10	8	1.4	4	8	9	8	2	0.07	0.04	0,4
F18-2-5	M	05-09-14	10	8	0.04	0.16	0.16	0.07	8	0.6	0.4	0.4	2	10	0.16	10
F19-3-2	F	08-04-15	10	10	10	8	4	8	2	2	8	4	10	2	4	4
F19-3-5	M	08-04-15	10	10	8	1.4	2	2	1.4	0.16	0.16	0.07	1.4	2	8	2

Supplementary Table S1 – Periorbital mechanical thresholds of F18 and F19 STA rats. Values indicated underneath column titles 'Day 1-Day 14' represent periorbital withdrawal threshold values (g) obtained manually with von Frey monofilaments over a 2 week period in two generations (F18 and F19) of adult STA rats. The F19 rats were supplied by the Thomas Jefferson University and used for subsequent breeding purposes within the animal facilities at the Danish Headache Center. Note that periorbital thresholds in all four rats appear to fluctuate episodically in agreement with Oshinsky et al., 2012. This differs with the data presented in Figure 1b and Figure 2a, b for subsequent generations of F1/F2 and F3 STA rats bred in house where all rats displayed a consistently low periorbital threshold compared with corresponding control SD rats.

Reference

1. Butcher, R.L., Collins, W.E. & Fugo, N.W. Plasma concentration of LH, FSH, prolactin, progesterone and estradiol-17beta throughout the 4-day estrous cycle of the rat. *Endocrinology* **94,** 1704-1708 (1974).
